# Correction to: A deficiency screen of the 3rd chromosome for dominant modifiers of the Drosophila ER integral membrane protein, Jagunal

**DOI:** 10.1093/g3journal/jkad212

**Published:** 2023-09-23

This is a correction to: Gerson Ascencio, Matthew A de Cruz, Judy Abuel, Sydney Alvarado, Yuma Arriaga, Emily Conrad, Alonso Castro, Katharine Eichelberger, Laura Galvan, Grace Gundy, Jorge Alberto Inojoza Garcia, Alyssa Jimenez, Nhein Tuyet Lu, Catharine Lugar, Ronald Marania, Tserendavaa Mendsaikhan, Jose Ortega, Natasha Nand, Nicole S Rodrigues, Khayla Shabazz, Cynnie Tam, Emmanuel Valenciano, Clive Hayzelden, Anthony S Eritano, Blake Riggs, A deficiency screen of the 3rd chromosome for dominant modifiers of the Drosophila ER integral membrane protein, Jagunal, *G3 Genes|Genomes|Genetics*, Volume 13, Issue 7, July 2023, jkad059, https://doi.org/10.1093/g3journal/jkad059

In the originally published version of this manuscript, a student co-author’s name was misspelled. Nhein Tuyet Lu should be correctly spelled Nhien Tuyet Lu.

This error has been corrected online.

