# Supplementary material for: A deficiency screen of the 3rd chromosome for dominant modifiers of the Drosophila ER integral membrane protein, Jagunal
Source: G3 (Bethesda). 2023 Mar 18;13(7):jkad059. doi: 10.1093/g3journal/jkad059 (PMC10320142; doi:10.1093/g3journal/jkad059)
Supplement: jkad059_Supplementary_Data [file jkad059_supplementary_data.zip › Supplemental_Material_Legends_G3-2022-403897.docx]

**Supplemental Figures and Tables**

**Supplemental Figure 1. Relative mRNA expression of Jagunal.**

RNA was isolated and qRT-PCR was used to quantify the relative mRNA levels of the gene Jagunal. Flies containing the UAS-Jagn RNAi with the Eyeless-GAL4 (Ey-GAL4) driver were used to measure the mRNA expression. Jagunal RNAi inhibits Jagn transcripts, thereby reducing the mRNA expression of Jagunal. Data is mean ± SEM. Significance was determined by a one-tailed test (* p-value < 0.05).

**Supplemental Figure 2. Modification of JagnRNAi scutellum phenotype.** UAS-JagnRNAi line was crossed with the P{GawB}^455.2^ line which expresses GAL4 in the scutellum region. (B) Expression of JagnRNAi in the scutellum region displayed defects in bristle formation including missing and shortened bristles (arrows). (C) JagnRNAi expression in unision with the deficiency BSC419 showed a suppression of the bristle phenotype with all four bristles being present. (D) Jagn RNAi expression with the dally mutation also displayed a supression of the bristle phenotype, with bristles being shorter similar to JagnRNAi expression (arrows) but there is a lack of missing bristles. These results in the scutellum are similar to results of BSC419 and dally suppression that was seen on the JagnRNAi rough eye phenotype.

**Supplemental Table 1. List of interested genes from deficiency modifiers.** Several deficiencies screened displayed either an enhancement or suppression of the JagnRNAi rough eye phenotype. Examination of genes covered by the deficiencies produced several potential genes that interact with Jagn. Genes were identified based on known biological function provided by Flybase and areas that did not overlap with deficiencies that did not display a modification.

**Supplemental Table 2. List of Drosophila stocks used.** In addition to Deficiency (DF) collection covering the 3^rd^ chromosome, there were several other stocks used including additional deficiencies listed that assisted in identifying gene targets due to their overlay with deficiencies found in the kit.
