# Supplementary material for: A deficiency screen of the 3rd chromosome for dominant modifiers of the Drosophila ER integral membrane protein, Jagunal
Source: G3 (Bethesda). 2023 Mar 18;13(7):jkad059. doi: 10.1093/g3journal/jkad059 (PMC10320142; doi:10.1093/g3journal/jkad059)
Supplement: jkad059_Supplementary_Data [file jkad059_supplementary_data.zip › Table_S1_G3-2022-403897.docx]

| **Table S1** | | |
| --- | --- | --- |
| **List of interested genes from deficiency modifiers.** | | |
| **Deficiency** | **Gene of interest** | **GO Annotations** |
| **3^rd^ left chromosome** | |  |
| Df(3L)ED208 | dar1 | Transcription factor in the Kruppel-like factor family |
|  | Eip63E | Cyclin-dependent kinase |
|  | miR-282 | Enables mRNA 3'-UTR binding activity and mRNA base-pairing post-transcriptional repressor activity. |
| Df(3L)Excel8104 | Sec63 | ER resident protein |
| Df(3L)BSC23 | Girdin | Enable dynein and microtubule-binding activity. |
| Df(3L)BSC391 | α-Tubulin at 67C | Form heterodimers with β-tubulins to polymerize and form microtubules |
| Df(3L)ED4421 | Dally | Glycoprotein, acts as a co-receptor for growth factors and morphogens. |
|  | Klp67A | Kinesin-like protein, encodes a microtubule motor protein involved in chromosome congression |
| Df(3L)ED4470 | CG7394 | Probable component of the PAM complex, |
| Df(3L)Exel6058 | miR-ban | Enables mRNA 3'-UTR binding activity and mRNA base-pairing post-transcriptional repressor activity. |
| Df(3L)ED4710 | Ccn | Enable heparin binding activity and integrin binding activity |
| Df(3L)BSC800 | α-COP | Part of the COPI coat complex involved in retrograde transport and is located at the ER-Golgi intermediate complex (ERGIC) |
| Df(3L)BSC839 | Presenilin (Psn) | Catalytic subunit of the gamma-secretase complex, an endoprotease complex that catalyzes the intramembrane cleavage of integral membrane proteins such as Notch receptor. |
| **3^rd^ Right chromosome** | |  |
| Df(3R)Excel6272 | Sec15 | Component of the exocyst complex involved in the docking of exocytic vesicles with fusion sites on the plasma membrane. |
|  | Rab11 | encodes a Rab type protein, Rab proteins are ubiquitously expressed family of small monomeric Ras-like GTPases that are essential for maintaining various cellular functions. |
| Df(3R)Exel6270 | Arl6IP1 | Involved in endoplasmic reticulum tubular network membrane organization |
