# Supplementary material for: A deficiency screen of the 3rd chromosome for dominant modifiers of the Drosophila ER integral membrane protein, Jagunal
Source: G3 (Bethesda). 2023 Mar 18;13(7):jkad059. doi: 10.1093/g3journal/jkad059 (PMC10320142; doi:10.1093/g3journal/jkad059)
Supplement: jkad059_Supplementary_Data [file jkad059_supplementary_data.zip › Table_S2_G3-2022-403897.docx]

| **Table S2. List of Drosophila stocks used.** | | |
| --- | --- | --- |
| Genotype | Source | Identifier |
| Oregon-R-P2 (Control) | Bloomington Drosophila Stock Center | 2376 |
| UAS-Jagn-RNAi | Vienna Drosophila Resource Center | 108991 |
| w[1118]; P{w[+mW.hs]=GawB}455.2, P{ry[+t7.2]=UAS-lacZ.Hsp70}2/CyO | Bloomington Drosophila Stock Center | 6820 |
| w[*]; P{w[+m*]=GAL4-ey.H}4-8/CyO | Bloomington Drosophila Stock Center | 5535 |
| w[1118]; Df(3L)ED50002, P{w[+mW.Scer\FRT.hs3]=3'.RS5+3.3'}ED50002 | Bloomington Drosophila Stock Center | 24627 |
| w[1118]; Df(3L)ED201, P{w[+mW.Scer\FRT.hs3]=3'.RS5+3.3'}ED201/TM6C, cu[1] Sb[1] | Bloomington Drosophila Stock Center | 8047 |
| w[1118]; Df(3L)ED4177, P{w[+mW.Scer\FRT.hs3]=3'.RS5+3.3'}ED4177/TM6C, cu[1] Sb[1] | Bloomington Drosophila Stock Center | 8048 |
| w[1118]; Df(3L)Exel6085, P{w[+mC]=XP-U}Exel6085/TM6B, Tb[1] | Bloomington Drosophila Stock Center | 7564 |
| w[1118]; Df(3L)BSC800, P+PBac{w[+mC]=XP3.WH3}BSC800/TM6C, Sb[1] cu[1] | Bloomington Drosophila Stock Center | 27372 |
| w[1118]; Df(3L)BSC181/TM6C, Sb[1] cu[1] | Bloomington Drosophila Stock Center | 9693 |
| w[1118]; Df(3L)ED4287, P{w[+mW.Scer\FRT.hs3]=3'.RS5+3.3'}ED4287/TM6C, cu[1] Sb[1] | Bloomington Drosophila Stock Center | 8096 |
| Df(3L)BSC23, rho[ve-1] e[1]/TM2, p[p] | Bloomington Drosophila Stock Center | 6755 |
| w[1118]; Df(3L)BSC672, P+PBac{w[+mC]=XP3.WH3}BSC672/TM6C, Sb[1] cu[1] | Bloomington Drosophila Stock Center | 26524 |
| w[1118]; Df(3L)ED4293, P{w[+mW.Scer\FRT.hs3]=3'.RS5+3.3'}ED4293/TM6C, cu[1] Sb[1] | Bloomington Drosophila Stock Center | 8058 |
| w[1118]; Df(3L)ED208, P{w[+mW.Scer\FRT.hs3]=3'.RS5+3.3'}ED208/TM6C, cu[1] Sb[1] | Bloomington Drosophila Stock Center | 8059 |
| w[1118]; Df(3L)BSC368/TM6C, Sb[1] cu[1] | Bloomington Drosophila Stock Center | 24392 |
| w[1118]; Df(3L)ED4341, P{w[+mW.Scer\FRT.hs3]=3'.RS5+3.3'}ED4341/TM6C, cu[1] Sb[1] | Bloomington Drosophila Stock Center | 8060 |
| w[1118]; Df(3L)ED210, P{w[+mW.Scer\FRT.hs3]=3'.RS5+3.3'}ED210/TM6C, cu[1] Sb[1] | Bloomington Drosophila Stock Center | 8061 |
| w[1118]; Df(3L)BSC371/TM6C, Sb[1] cu[1] | Bloomington Drosophila Stock Center | 24395 |
| w[1118]; Df(3L)BSC884/TM6C, Sb[1] cu[1] | Bloomington Drosophila Stock Center | 30589 |
| w[1118]; Df(3L)BSC410/TM6C, Sb[1] cu[1] | Bloomington Drosophila Stock Center | 24914 |
| w[1118]; Df(3L)Exel6109, P{w[+mC]=XP-U}Exel6109/TM6B, Tb[1] | Bloomington Drosophila Stock Center | 7588 |
| Df(3L)BSC27/TM6B, Tb[1] | Bloomington Drosophila Stock Center | 6867 |
| w[1118]; Df(3L)BSC117/TM6B, Tb[1] | Bloomington Drosophila Stock Center | 8974 |
| w[1118]; Df(3L)BSC33, rho[ve-1]/TM3, Sb[1] | Bloomington Drosophila Stock Center | 6964 |
| w[1118]; Df(3L)Exel8104/TM6B, Tb[1] | Bloomington Drosophila Stock Center | 7929 |
| w[1118]; Df(3L)BSC875, P+PBac{w[+mC]=XP3.WH3}BSC875/TM6C, Sb[1] cu[1] | Bloomington Drosophila Stock Center | 30580 |
| w[1118]; Df(3L)BSC388/TM6C, Sb[1] cu[1] | Bloomington Drosophila Stock Center | 24412 |
| w[1118]; Df(3L)Exel6112, P{w[+mC]=XP-U}Exel6112/TM6B, Tb[1] | Bloomington Drosophila Stock Center | 7591 |
| w[1118]; Df(3L)BSC815, P+PBac{w[+mC]=XP3.WH3}BSC815/TM6C, Sb[1] cu[1] | Bloomington Drosophila Stock Center | 27576 |
| w[1118]; Df(3L)BSC389/TM6C, Sb[1] cu[1] | Bloomington Drosophila Stock Center | 24413 |
| w[1118]; Df(3L)BSC816, P+PBac{w[+mC]=XP3.WH3}BSC816/TM6C, Sb[1] cu[1] | Bloomington Drosophila Stock Center | 27577 |
| w[1118]; Df(3L)ED4421, P{w[+mW.Scer\FRT.hs3]=3'.RS5+3.3'}ED4421/TM6C, cu[1] Sb[1] | Bloomington Drosophila Stock Center | 8066 |
| w[1118]; Df(3L)BSC113/TM6B, Tb[1] | Bloomington Drosophila Stock Center | 8970 |
| w[1118]; Df(3L)BSC391/TM6C, Sb[1] cu[1] | Bloomington Drosophila Stock Center | 24415 |
| w[1118]; Df(3L)BSC392/TM6C, Sb[1] cu[1] | Bloomington Drosophila Stock Center | 24416 |
| w[1118]; Df(3L)BSC673, P+PBac{w[+mC]=XP3.WH3}BSC673/TM6C, Sb[1] cu[1] | Bloomington Drosophila Stock Center | 26525 |
| w[1118]; Df(3L)ED4457, P{w[+mW.Scer\FRT.hs3]=3'.RS5+3.3'}ED4457/TM6C, cu[1] Sb[1] | Bloomington Drosophila Stock Center | 9355 |
| w[1118]; Df(3L)ED4470, P{w[+mW.Scer\FRT.hs3]=3'.RS5+3.3'}ED4470/TM6C, cu[1] Sb[1] | Bloomington Drosophila Stock Center | 8068 |
| w[1118]; Df(3L)ED4475, P{w[+mW.Scer\FRT.hs3]=3'.RS5+3.3'}ED4475/TM6C, cu[1] Sb[1] | Bloomington Drosophila Stock Center | 8069 |
| w[1118]; Df(3L)BSC730/TM6C, Sb[1] cu[1] | Bloomington Drosophila Stock Center | 26828 |
| w[1118]; Df(3L)ED4486, P{w[+mW.Scer\FRT.hs3]=3'.RS5+3.3'}ED4486/TM6C, cu[1] Sb[1] | Bloomington Drosophila Stock Center | 8072 |
| Df(3L)BSC12, rho[ve-1] e[1]/TM3, P{w[+m*]=Ubx-lacZ.w[+]}TM3, Sb[1] | Bloomington Drosophila Stock Center | 6457 |
| w[1118]; Df(3L)ED4502, P{w[+mW.Scer\FRT.hs3]=3'.RS5+3.3'}ED4502/TM6C, cu[1] Sb[1] | Bloomington Drosophila Stock Center | 8097 |
| w[1118]; Df(3L)ED4543, P{w[+mW.Scer\FRT.hs3]=3'.RS5+3.3'}ED4543/TM6C, cu[1] Sb[1] | Bloomington Drosophila Stock Center | 8073 |
| w[1118]; Df(3L)ED217, P{w[+mW.Scer\FRT.hs3]=3'.RS5+3.3'}ED217/TM6C, cu[1] Sb[1] | Bloomington Drosophila Stock Center | 8074 |
| w[1118]; Df(3L)BSC845/TM6C, Sb[1] cu[1] | Bloomington Drosophila Stock Center | 27888 |
| w[1118]; Df(3L)BSC774/TM6C, Sb[1] cu[1] | Bloomington Drosophila Stock Center | 27346 |
| w[1118]; Df(3L)ED4606, P{w[+mW.Scer\FRT.hs3]=3'.RS5+3.3'}ED4606/TM6C, cu[1] Sb[1] | Bloomington Drosophila Stock Center | 8078 |
| w[1118]; Df(3L)ED4674, P{w[+mW.Scer\FRT.hs3]=3'.RS5+3.3'}ED4674/TM6C, cu[1] Sb[1] | Bloomington Drosophila Stock Center | 8098 |
| w[1118]; Df(3L)BSC414/TM6C, Sb[1] cu[1] | Bloomington Drosophila Stock Center | 24918 |
| w[1118]; Df(3L)ED4710, P{w[+mW.Scer\FRT.hs3]=3'.RS5+3.3'}ED4710/TM3, Sb[1] | Bloomington Drosophila Stock Center | 8100 |
| w[1118]; Df(3L)BSC775/TM6C, Sb[1] cu[1] | Bloomington Drosophila Stock Center | 27347 |
| w[1118]; Df(3L)BSC220/TM6C, Sb[1] cu[1] | Bloomington Drosophila Stock Center | 9697 |
| w[1118]; Df(3L)ED229, P{w[+mW.Scer\FRT.hs3]=3'.RS5+3.3'}ED229/TM6C, cu[1] Sb[1] | Bloomington Drosophila Stock Center | 8087 |
| w[1118]; Df(3L)ED4858, P{w[+mW.Scer\FRT.hs3]=3'.RS5+3.3'}ED4858/TM2 | Bloomington Drosophila Stock Center | 8088 |
| w[1118]; Df(3L)BSC839/TM6C, Sb[1] cu[1] | Bloomington Drosophila Stock Center | 27917 |
| w[1118]; Df(3L)BSC797/TM6C, Sb[1] cu[1] | Bloomington Drosophila Stock Center | 27369 |
| w[1118]; Df(3L)BSC449/TM6C, Sb[1] cu[1] | Bloomington Drosophila Stock Center | 24953 |
| w[1118]; Df(3L)BSC553/TM6C, Sb[1] | Bloomington Drosophila Stock Center | 25116 |
| w[1118]; Df(3L)BSC419/TM6C, Sb[1] cu[1] | Bloomington Drosophila Stock Center | 24923 |
| w[1118]; Df(3L)ED4978, P{w[+mW.Scer\FRT.hs3]=3'.RS5+3.3'}ED4978/TM6C, cu[1] Sb[1] | Bloomington Drosophila Stock Center | 8101 |
| w[1118]; Df(3L)BSC249/TM6C, Sb[1] cu[1] | Bloomington Drosophila Stock Center | 23149 |
| w[1118]; Df(3L)ED230, P{w[+mW.Scer\FRT.hs3]=3'.RS5+3.3'}ED230/TM6C, cu[1] Sb[1] | Bloomington Drosophila Stock Center | 8089 |
| w[1118]; Df(3L)ED5017, P{w[+mW.Scer\FRT.hs3]=3'.RS5+3.3'}ED5017/TM6C, cu[1] Sb[1] | Bloomington Drosophila Stock Center | 8102 |
| Df(3L)TTT, P{y[+mDint2] w[BR.E.BR]=SUPor-P}KG03264 ry[506]/TM3, Sb[1] | Bloomington Drosophila Stock Center | 81882 |
| Df(3L)6B-29+Df(3R)6B-29, kni[ri-1] p[p]/TM3, Ser[1] | Bloomington Drosophila Stock Center | 2596 |
| w[1118]; Df(3R)Exel6272, P{w[+mC]=XP-U}Exel6272/TM6B, Tb[1] | Bloomington Drosophila Stock Center | 7739 |
| Df(3R)Ubx109/Dp(3;3)P5 | Bloomington Drosophila Stock Center | 3486 |
| w[1118]; Df(3R)BSC887/TM6B, Tb[+] | Bloomington Drosophila Stock Center | 30592 |
| Df(3R)BSC43, st[1] ca[1]/TM2, p[p] | Bloomington Drosophila Stock Center | 7413 |
| w[1118]; Df(3R)ED10639, P{w[+mW.Scer\FRT.hs3]=3'.RS5+3.3'}ED10639/TM6C, cu[1] Sb[1] | Bloomington Drosophila Stock Center | 9481 |
| w[1118]; Df(3R)BSC650/TM6C, Sb[1] cu[1] | Bloomington Drosophila Stock Center | 25740 |
| w[1118]; Df(3R)BSC819, P+PBac{w[+mC]=XP3.RB5}BSC819/TM6C, Sb[1] cu[1] | Bloomington Drosophila Stock Center | 27580 |
| w[1118]; Df(3R)BSC790, P+PBac{w[+mC]=XP3.WH3}BSC790/TM6C, Sb[1] cu[1] | Bloomington Drosophila Stock Center | 27362 |
| w[1118]; mir-282[RSX] e[s]/TM3, Sb[1] Ser[1] | Bloomington Drosophila Stock Center | 52667 |
| w[*]; Eip63E[81]/TM6B, Tb[1] | Bloomington Drosophila Stock Center | 4513 |
| w[1118]; PBac{w[+mC]=IT.GAL4}ntc[0521-G4] CG32264[0521-G4]/TM6B, Tb[1] | Bloomington Drosophila Stock Center | 63376 |
| w[1118]; PBac{w[+mC]=PB}Girdin[c06007]/TM6B, Tb[1] | Bloomington Drosophila Stock Center | 85068 |
| w[1118]; PBac{w[+mC]=WH}dar1[f06798]/TM6B, Tb[1] | Bloomington Drosophila Stock Center | 86113 |
| w[1118]; PBac{w[+mC]=RB}Sec63[e03550]/TM6B, Tb[1] | Bloomington Drosophila Stock Center | 85580 |
| alphaTub67C[1] kni[ri-1] e[1]/TM3, Sb[1] | Bloomington Drosophila Stock Center | 1750 |
| w[1118]; PBac{w[+mC]=WH}dally[f01984] | Bloomington Drosophila Stock Center | 18500 |
| w[1118]; PBac{w[+mC]=PB}CG7394[c04985]/TM6B, Tb[1] | Bloomington Drosophila Stock Center | 17682 |
| y[1] w[1118]; P{w[+mC]=lacW}mir-ban[L1170a] lncRNA:CR43334[L1170a], l(3)L1170b[L1170b]/TM3, Ser[1] | Bloomington Drosophila Stock Center | 10154 |
| w[1118]; PBac{w[+mC]=WH}Ccn[f03521] | Bloomington Drosophila Stock Center | 18665 |
| y[d2] w[1118] P{ry[+t7.2]=ey-FLP.N}2 P{5xglBS-lacZ.38-1}TPN1; P{ry[+t7.2]=neoFRT}82B Sec15[1]/TM3, P{w[+mC]=GAL4-Kr.C}DC2, P{w[+mC]=UAS-GFP.S65T}DC10, Sb[1] | Bloomington Drosophila Stock Center | 24889 |
| y[1] w[*]; P{w[+mC]=lacW}Rab11[j2D1]/TM3, Sb[1] | Bloomington Drosophila Stock Center | 12148 |
| w[*]; P{w[+mC]=EP}Klp67A[322b24]/TM6B, Tb[1] | Bloomington Drosophila Stock Center | 35507 |
| y[1] v[1]; P{y[+t7.7] v[+t1.8]=TKO.GS04824}attP40 | Bloomington Drosophila Stock Center | 81494 |
| y[1] v[1]; P{y[+t7.7] v[+t1.8]=TKO.GS05524}attP2 | Bloomington Drosophila Stock Center | 84060 |
| w[1118]; Psn[143]/TM6B, Tb[1] | Bloomington Drosophila Stock Center | 8297 |
| Psn[C4]/TM6C, Sb[1] Tb[1] | Bloomington Drosophila Stock Center | 63238 |
