# Supplementary figures and images for: A deficiency screen of the 3rd chromosome for dominant modifiers of the Drosophila ER integral membrane protein, Jagunal
Source: G3 (Bethesda). 2023 Mar 18;13(7):jkad059. doi: 10.1093/g3journal/jkad059 (PMC10320142; doi:10.1093/g3journal/jkad059)

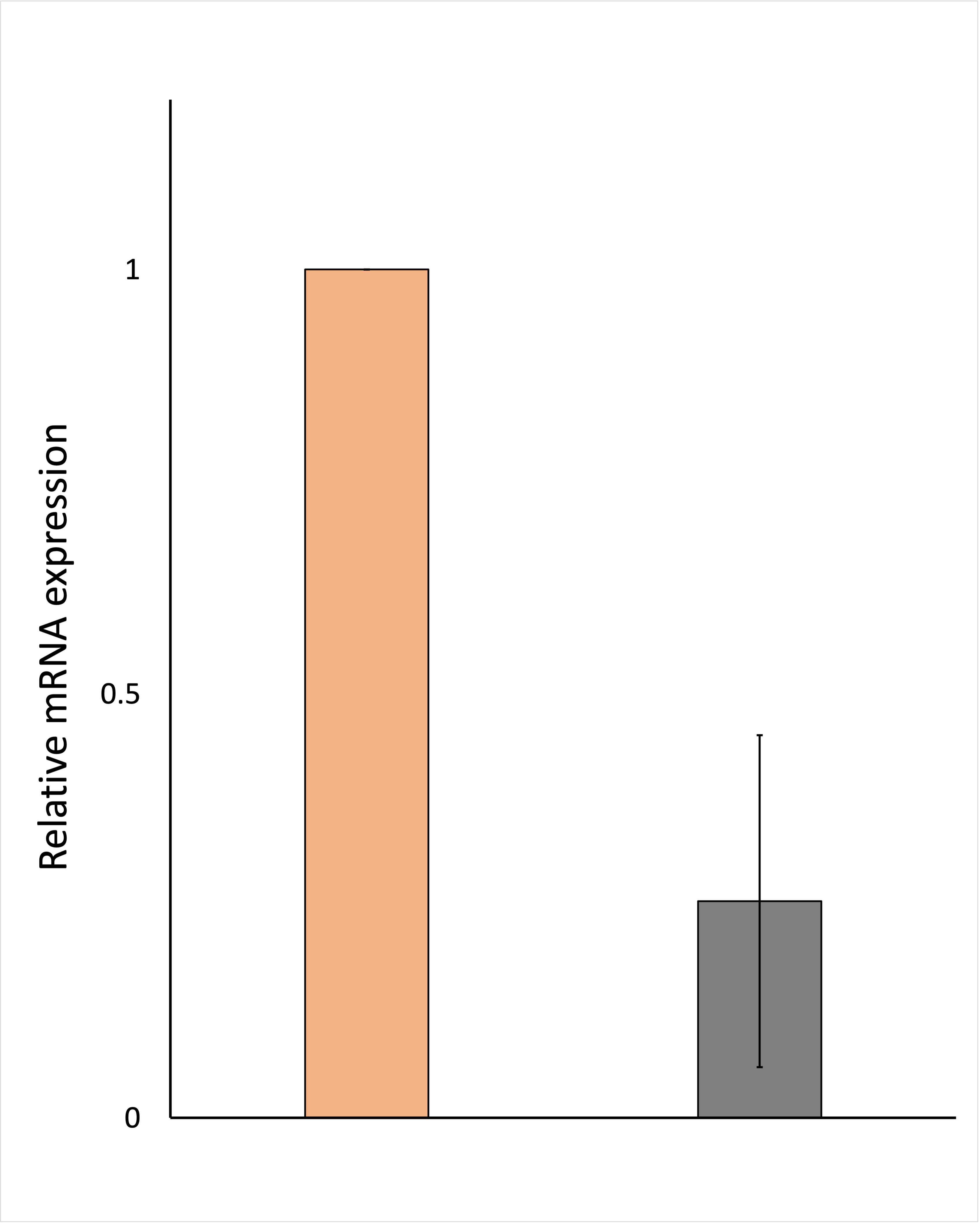

Supplement: jkad059_Supplementary_Data [file jkad059_supplementary_data.zip › Figure_S1_G3-2022-403897.tif]

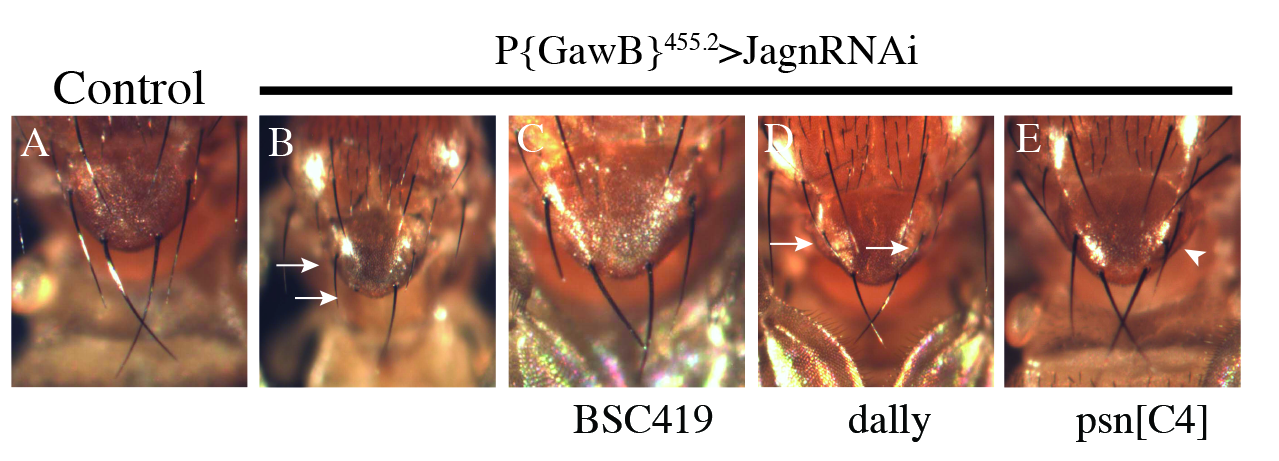

Supplement: jkad059_Supplementary_Data [file jkad059_supplementary_data.zip › Figure_S2_G3-2022-403897.tif]
